# Cultural attitudes and human pressure towards vultures around the Comoé National Park, Côte d’Ivoire (West Africa)

**DOI:** 10.1186/s13002-024-00657-0

**Published:** 2024-02-29

**Authors:** Asso Armel Asso, N’golo Abdoulaye Koné, Volker Salewski

**Affiliations:** 1 Laboratoire d’Ecologie et de Développement Durable (LEDD), UFR Sciences de la Nature (UFR SN), Université Nangui ABROGOUA, Abidjan, Côte d’Ivoire; 2 Station de Recherche en Écologie du Parc National de La Comoé, 28 BP 847 Abidjan 28, Côte d’Ivoire; 3 Michael-Otto-Institut Im NABU, Goosstroot 1, 24861 Bergenhusen, Germany

**Keywords:** Vultures, Ethno-ornithology, Magico-traditional medicine, Comoé National Park, Côte d’Ivoire

## Abstract

**Background:**

Populations of vultures in Africa have experienced a rapid decline over recent decades, with some species suffering losses of more than 90%. Various forms of human pressures have been identified as the causes of this decline. However, very little is known about the complex interaction between cultural practises, traditional medicine and the vultures’ natural environment. The purpose of this study was to analyse human pressures on vultures in relation with cultural attitudes such as their demand for magico-traditional medicine in Côte d’Ivoire, around the Comoé National Park (CNP), one of the last major refuges of these organisms in West Africa.

**Methods:**

Eleven surrounding villages were visited to carry out ethno-ornithology surveys. One hundred and ten people were interviewed, at a rate of ten people per village, using a semi-structured questionnaire and informal discussions.

**Results:**

The findings showed that vultures are still being seen both in and around the CNP. The most common species indicated to be observed and indicated by the interviewees were the Hooded Vulture (*Necrosyrtes monachus*) and the White-headed Vulture (*Trigonoceps occipitalis*). Nevertheless, 98.2% of the interviewees indicated having observed a decrease in the abundance of vultures over the last few years in the study area, as well as a decline in the number of sightings of these organisms. Interviewees attributed this scarcity of vultures to (1) the limited availability of food resources, (2) pesticide and tobacco poisoning, (3) hunting, (4) rapid population growth, (5) annual bushfires and (6) habitat loss. The uses of the vulture or parts of vultures by the populations surrounding the CNP (traditional medicine, mystic practises, consumption as food, cultural heritage) were also highlighted as real threats to these organisms. And the vulture parts commonly used in this area are: feathers, legs, head, heart, stomach, brain and excrement. The Hooded Vulture (*Necrosyrtes monachus*) appears to be the most sought-after species and the most widely used for these practises in the study area.

**Conclusion:**

Appropriate conservation and communication initiatives are required to ensure the survival of these raptors, crucial for ecosystem well-being, while also ensuring a respect of cultural practises.

**Supplementary Information:**

The online version contains supplementary material available at 10.1186/s13002-024-00657-0.

## Background

Vultures have declined dramatically in Africa over the past decades and are at the brink of extinction in many regions [1, 2]. The decline is especially pronounced in West Africa, where it was already recognised locally some decades ago [3, 4]. The reasons for the decline are complex, but besides reduced availability of food, habitat degradation with lack of nesting trees, electrocution and collision with electrical infrastructure [1, 2] intentional persecution such as poisoning is responsible for the collapse of many vulture populations throughout West Africa [5–8]. A drastic recent example is the discovery of more than 2000 killed Hooded Vultures (*Necrosyrtes monachus* Temminck, 1823) in Guinea Bissau in 2019/20 [9].

All vulture species occurring in West Africa are listed in the Bonn Convention of Migratory Species (CMS-Convention) and in Appendix II of the Convention on International Trade in Endangered Species (CITES-Convention). Among the species mentioned are: White-backed Vulture (*Gyps africanus* Salvadori, 1865), Rüppell’s Vulture (*Gyps rueppelli* Brehm, 1852), White-headed Vulture (*Trigonoceps occipitalis* Burchell, 1824) and Hooded Vulture (*Necrosyrtes monachus*) which are Critically Endangered (CR) and Lappet-faced Vulture (*Torgos tracheoliotus* Forster, 1791) endangered (EN). Even though vultures are, therefore, legally protected in West African signatory countries like Côte d’Ivoire, the driver for the unsustainable active persecution is the widespread trade of vultures and their parts for traditional belief-based use [6, 10–12]. Furthermore, in this African region, there is evidence for trans-border trade of vultures or their body parts [10, 11, 13–16], with trade for belief-based use as the main threat for local vulture populations [8, 15–17].

In Côte d’Ivoire systematically country-wide collected data on the abundance and distribution of vultures in the past are missing. There are, however, reports that Hooded Vulture (*Necrosyrtes monachus*), which is often commensal with humans [18], declined in the southern and central parts of the country already in the 1950s and 1960s [3, 19]. Information about the former status of species like Lappet-faced Vulture (*Torgos tracheliotus*), White-backed Vulture (*Gyps africanus*) and White-headed Vulture (*Trigonoceps occipitalis*) is almost non-existent. It can, however, be assumed that these species were once widespread in the northern savannahs of the country and, at least in the case of the White-backed Vulture, also abundant [20, 21]. Now, Hooded Vulture is gone from almost all villages and only single individuals are a rare sight at some slaughterhouses with recent observations from Abengourou in the east and Bouna in the north–east (own unpublished observations). Other species are probably restricted to large reserves with Comoé National Park in the north-east of the country being probably the last region where Hooded Vulture, White-backed Vultures and White-headed Vultures are breeding regularly [22] although systematic surveys in other reserves are missing.

Protected areas as refuges for threatened species play a key role in vulture-conservation in West Africa [7, 14, 23]. Even though the latter may be last strongholds for most species [7, 23], declines have also been reported in many of them [14, 23, 24]. The reason is most likely direct persecution to provide West African markets with vulture parts for the demand connected to traditional belief-based use [8, 16, 17] as reported from several countries in the region such as Benin [13], Burkina Faso [16], Ghana [17, 25] and Nigeria [8, 26]. However, with respect to Côte d’Ivoire, very little is known about the extent and importance of poaching and trade in vultures especially around remote protected areas and their roles for local economy and culture.

Local understanding and perceptions of wildlife must be given special attention when implementing conservation strategies for threatened species [27–30]. Therefore, the Abrogoua-Nangui University of Abidjan (Côte d’Ivoire) in co-operation with the NABU International Foundation for Nature, (Germany), started an ethno-ornithology survey to analyse the cultural considerations of vultures by the inhabitants of villages around Comoé National Park and to assess the human pressure generated by the demand for vultures for traditional belief-based use.

## Methods

### Comoé National Park

**Fig. 1:**
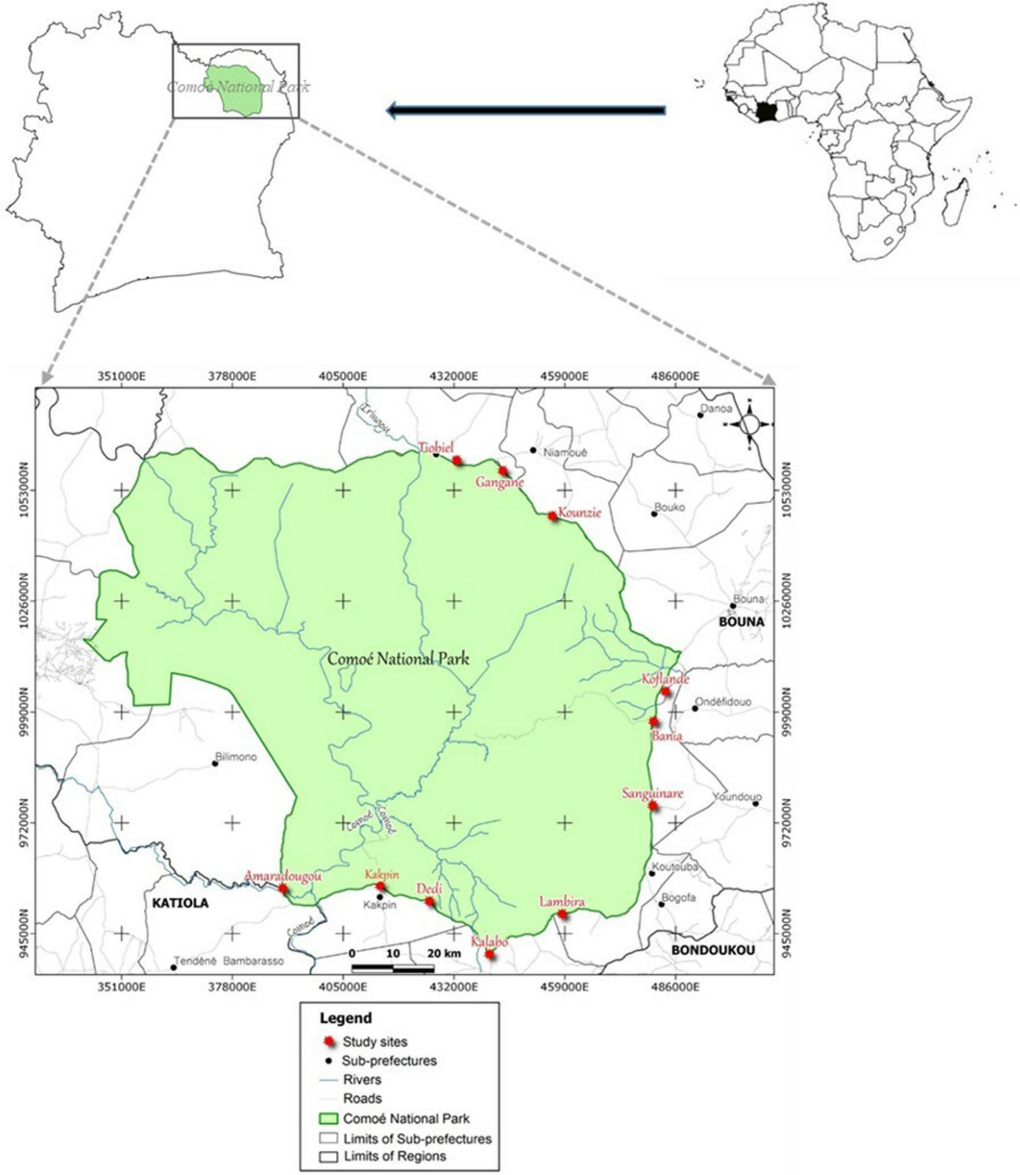
Villages on the periphery of the Comoé National Park visited during the study

The Comoé National Park (CNP) is one of the largest National Parks in West Africa and is situated in north-east Côte d’Ivoire (Fig. 1) on the transition between the Northern Guinean and the Sudanian Savannah zones. It was created in 1953 and declared a National Park in 1958 by presidential decree [31]. The CNP is listed as a Biosphere Reserve and World Heritage Site and covers c. 11,500 km^2^. The CNP extends from about 8.5 to 9.5° N and from about 3.0 to 4.5° W. The 100–200 m-wide Comoé River flows north–south through CNP for about 220 km and drains most of it [31]. Tree and bush savannah cover about 70% of CNP [31]. Gallery forests are found along the Comoé River and its larger tributaries. Forest patches of various sizes also occur as scattered islands through the savannah, especially in the south [32]. Five species of vultures have been recorded in the park, namely the Palm-nut Vulture (*Gypohierax angolensis* Gmelin, 1788), the Hooded Vulture (*Necrosyrtes monachus*), the White-backed Vulture (*Gyps africanus*), the Lappet-faced Vulture (*Torgos tracheliotus*) and the White-headed Vulture (*Trigonoceps occipitalis*) [33]. The Hooded, White-backed Vulture and White-headed Vulture breed in the dry season (November–March) and almost exclusively in gallery forests and forest islands. Nests are almost exclusively in large Kapok (*Ceiba pentandra*) trees, which grow only in these forests [22]. Rüppell’s Vulture (*Gyps rueppelli*) has been recorded in the north of the country [19]. Although never explicitly reported from the CNP [33] (Salewski 2000), this species may also occur.

### Human population around the Comoé National Park

Almost 300,000 people inhabit the periphery of the CNP with a mean population density of 18.4 inhabitants/km^2^. The highest population densities are found at the eastern fringe of the park, whereas the north–western, the south–western and especially the western fringes of the park are less densely populated [34]. The main ethnics found around the CNP are Lobi, Koulango, Djimini and Malinké, the predominant religions are Islam, Christianity and animistic religions [34]. There is no legal settlement within the park boundaries, but there are illegal camps of poachers, fishermen, pastoralists and goldminers of varying size scattered over the park and especially along the Comoé River [34, 35].

### Ethno-ornithology surveys

**Fig. 2:**
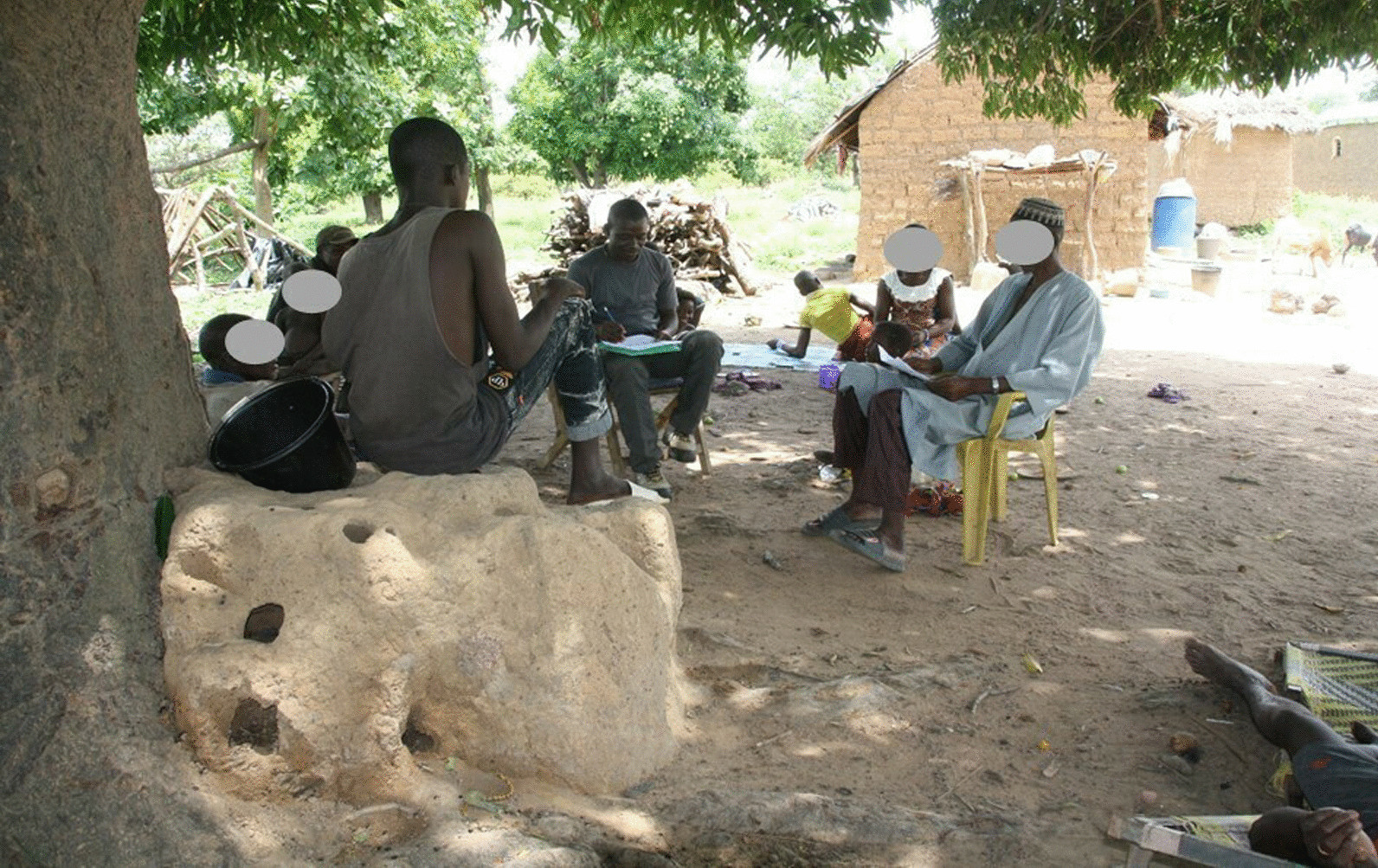
A. A. Asso interviewing villagers, Gangane 19 Mai 2021

For the ethno-zoological surveys on vultures, interviews were conducted between 08 May and 01 June 2021 in eleven villages around CNP. The villages were selected on the basis of accessibility, proximity to the park and village size, and were chosen to encircle the park as far as possible (Fig. 2). Approval to conduct the research came from Nangui ABROGOUA University, in agreement with the regional prefects and traditional authorities (village chiefs, land chiefs and youth presidents) in each study area. Selected villages had to be at least two kilometres apart from each other. The villages visited were: Amaradougou, Bania, Dédi, Gangane, Kakpin, Kalabo, Koflandé, Kounzié, Lambira, Sanguinaré and Tiobiel. They were situated on the southern, eastern and north-eastern limits of CNP (Fig. 1). Due to some security issues linked to terrorism in some neighbouring countries, no villages of the north-western part of the park were visited during these ethno-zoological surveys. Each village was visited only once to avoid replication and respondent bias in the answers. One to two days were spent in each village for data collection.

The interviewees were at least 18 years old and residents of the respective visited villages; so mature enough to provide information on their respective environment and the topic of the survey. Ten persons were randomly selected in each of the visited villages. So, for these ethno-zoological surveys, a total 110 persons were interviewed. Furthermore, in line with the British Sociological Association’s [36] Statement of Ethical Practice, informed consent was obtained verbally by each survey participant prior to the interview, participants were informed of their right to participate voluntarily or decline, no participant identification data were collected and the database collected was entirely anonymous. The interview started once the respondent agreed to participate in the study. The interviews were conducted either from 4:00 to 6:00 p.m. (interviewees returning from the fields) or from 8:00 to 12:00 a.m. during the local market days.

To determine respondents’ knowledge of vultures, photographs of the six vulture species occurring or potentially occurring in CNP (see above) were shown to the respondents [25]. The interviews were conducted in French and then translated into the local languages Koulango or Lobi depending on the level of education. To gain the trust from the respondents, the interviewer was accompanied by an assistant, native of the study area. Each interviewee was asked a set of predetermined open, close or multiple choice questions, using a semi-structured questionnaire [25].

### Data analysis

All data were stored and processed in an Excel 2016 spreadsheet.

Uses of vultures or their organs were assigned to four different use categories:

- Medicinal: e.g. use to make women fertile, cure blurred vision, earache, toothache, anaemia.
- Mystical: e.g. use to gain a lot of money, making one’s business prosper, to attract customers, protection against evil spells, having a long life, attracting good luck, acquiring supernatural strength, using to cast spells.
- Cultural: e.g. use of feathers to decorate hats.
- Alimentary: Use for food.

Several indices commonly applied in ethno-zoological and ethno-botanical studies were used to analyse the species-specific importance of use categories and the species-specific importance of certain organs as well as the consensus between the respondents about traits connected to their species-specific use in different use categories (Table 1). Chi-square analyses in R 4.2.1 software were used to test the relationships between the different variables.

**Table 1:** Indices calculated from the respondents’ answers about the use of the organs and excrements of vultures

Index	Calculation	Explanation and reference
Use value (UV)	UV = $\frac{{\sum {\text{SI}}}}{N}$ SI: a respondent who reported a specific use category for a given species *N*: the number of respondents	Identifies the species-specific relative importance of each use category mentioned by the respondents [37, 38]
Informant consensus factor (F_IC_)	*F*_IC_ = $\frac{{N_{{{\text{ur}}}} - N_{{\text{t}}} }}{{N_{{{\text{ur}}}} - 1}}$ *N*_ur_: number of use-reports for each usage category N_t_: number of usage categories mentioned by the responds	Identifies the level of consensus among the respondents about species-specific use categories; ranges between 0 and 1 and increased values indicate higher consensus [39]
Use diversity index (UD)	UD = $\frac{{U_{{{\text{cx}}}} }}{{U_{{{\text{ct}}}} }}$ *U*_cx_: number of records of a species within a use category *U*_ct_: sum of records of a species across all use categories	UD measures the importance of a species-specific use category and how evenly a species-specific use category contributes to its total use for a species [40]; ranges between 0 and the number of use categories for a species
Use equitability value (UE)	UE = $\frac{{{\text{UD}}}}{{{\text{UD}} _{{{\text{max}}}} }}$ UD: diversity of use for a given use category of a species UD_max_: maximum possible UD for a species with uses in a given number of categories	UE measures how evenly the different uses contribute to the total use of a species independent of the number of use categories [41]; ranges between 0 and 1; following Awo [42] we assume that knowledge of use is equal if UE ≥ 0.5, and unequal if UE < 0.5
Citation frequency (CF)	CF [%] = $\frac{n}{N}$ × 100 *n*: number of citations for a specific organ of a species *N*: total number of respondents mentioning a species to be used	CF measures the species-specific diversity of uses of certain organs and excrements of vultures [43]
Fidelity level (FL)	FL [%] = ${ }\frac{{N_{{\text{p}}} }}{N}$ × 100 *N*_p_: number of informants who mentioned a specific organ of a certain species to be used *N*: sum of citations of all organs to be used in different use categories	FL is the percentage of informants claiming the use of a certain organ [44]

## Results

### Characteristics of interviewees

The highest number of interviewees was over 60 years old (29.1%) followed by the age range of 41 to 50 years (see Additional file 1). The age range of 51–60 years contained 16.4% of the interviewees, while the one of 31–40 years represented 15.5% of them. The youngest age ranges (21 to 30 and < 21 years), respectively, accounted for 10.0% and 0.9% of the interviewees.

Most of the interviewees were male (80%, see Additional file 1). Almost all of the males were farmers (97.7%), whereas all interviewed women indicated being housewives.

With respect to religion, 51.8% (*n* = 57) of the interviewees were animists, 35.5% (*n* = 39) Muslims and 12.7% Christians (see Additional file 1).

There was a significant bias towards males (*χ*^2^1 = 39.6, *p* < 0.001), animists (*χ*^2^2 = 25.44, *p* < 0.001) and older age ranges (*χ*^2^5 = 38.36, *p* < 0.001), even if the only person under 21 years of age (*χ*^2^4 = 15.73, *p* = 0.003) was not considered.

### Knowledge about vulture species among the interviewees

**Fig. 3:**
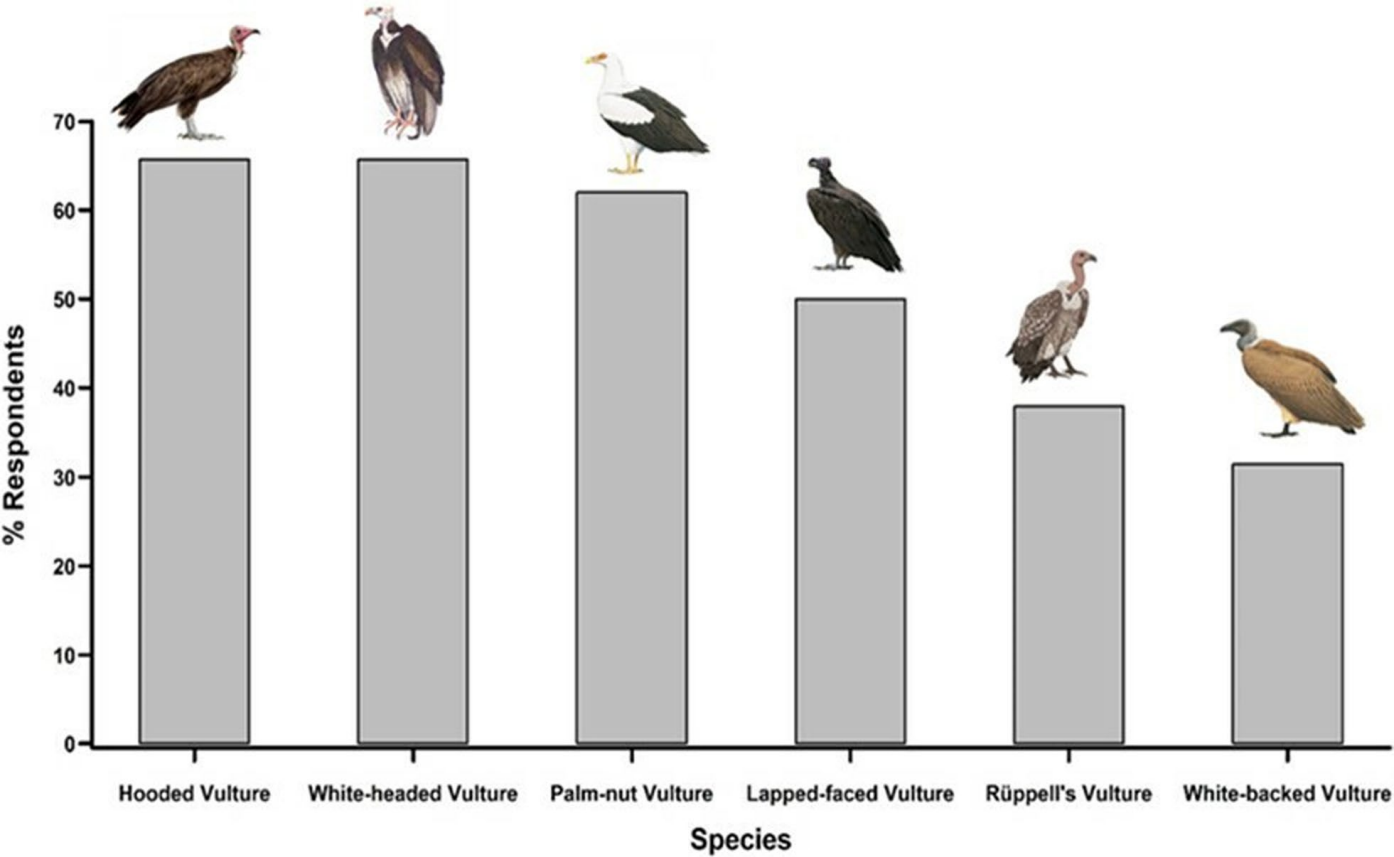
Respondents’ claimed knowledge of vulture species. A single respondent can indicate several species

Most of the interviewees (98.2%) were able to recognise at least one vulture species of the photographs. The Hooded Vulture and the White-headed Vulture were claimed to be the most known species by the interviewees (65.7%); followed by the Palm-nut Vulture (62.0%), the Lappet-faced Vulture (50.0%), the Rüppell’s Vulture (37.9%) and the White-backed Vulture (31.5%) (Fig. 3). Roughly half of the interviewees (49.1%) were able to give local names and their respective meaning to some vulture species (Table 2). Many of the respondents (48.2%) named species in local languages, but did not know the meaning of these names. Only three of the respondents (2.7%) had no idea of local names of vultures.

**Table 2:** Names of vultures in local languages according to locality

Locality	Vultures		
	Local name	Scientific name	Meaning of local names
Amaradougou	Douga or Tadjenkonan	*Trigonoceps occipitalis*	The king of vultures
		*Torgos tracheoliotus*	The hunting vulture
		*Gyps africanus*	The fire bird
Kakpin	Douga	*Necrosyrtes monachus*	The meat-eater
		*Gyps rueppelli*	The elephant piercer
	Doun	*Gypohierax angolensis*	The white one
Dédi	Douga	*Trigonoceps occipitalis*	The profiteer
		*Torgos tracheoliotus*	The malefactor
		*Gyps rueppelli*	The villain
	Kokosaki	*Gypohierax angolensis*	The seed-eater
Kalabo	Kokosaki	*Gypohierax angolensis*	The seed-eater
Lambira	Douga	*Trigonoceps occipitalis*	The malefactor
		*Gyps africanus*	The fire bird
		*Torgos tracheoliotus*	The hunting vulture
	Kokosaki	*Gypohierax angolensis*	The seed-eater
Sanguinaré	Doun	*Gypohierax angolensis*	The seed-eater
	Para or Kplé	*Trigonoceps occipitalis*	The corpse-eater
Koflandé	Douga	*Trigonoceps occipitalis*	The meat-eater
	Loméo	*Gypohierax angolensis*	The seed-eater
Kounzié	Poulo	*Torgos tracheoliotus*	The meat-eater
		*Trigonoceps occipitalis*	The meat-eater
Gangane	Doun	*Gypohierax angolensis*	The seed-eater
	Kplé or Para	*Trigonoceps occipitalis*	The corpse-eater
Tiobiel	Doun	*Gypohierax angolensis*	The seed-eater
Bania	Loméo	*Gypohierax angolensis*	The seed-eater
	Douga	*Necrosyrtes monachus*	The meat-eater

Local languages: Koulango, Lobi and Dioula

### Locations, periods and frequency of vulture observations in the localities visited

The CNP was identified as the main place where living vultures are encountered (*n* = 47; 42.7%). Some respondents (*n* = 16, 14.5%) also reported observing living vultures in around their respective villages, usually when a large animal died. The slaughterhouse in the city of Bouna (Fig. 1) was also identified by 11.8% of the interviewees (*n* = 13) as another place where living vultures are encountered during the dry season. Finally, according to 9.1% of the interviewees, living vultures are occasionally observed in their villages when the wild palm trees around these villages are fruiting.

**Fig. 4:**
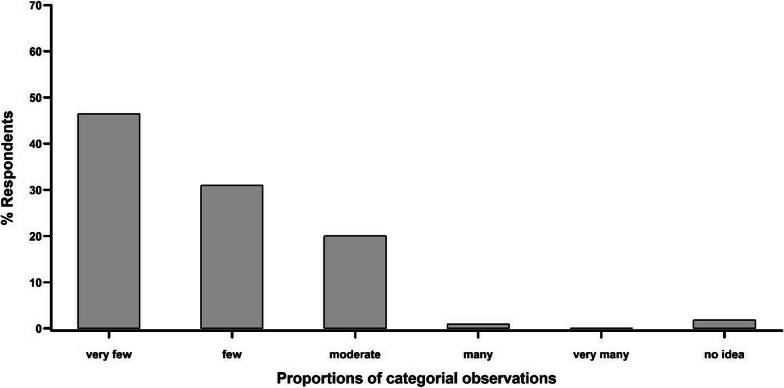
Categorial frequencies of vulture observations according to the respondents

Most of the respondents (98.2%) indicated to observe a decrease in vulture numbers over the last years. Most of them also mentioned that it was very difficult to observe live vultures in their locality today compared to the past (Fig. 4). In fact, none of the people interviewed reported seeing more vultures in recent years. A large proportion of interviewees (71.8%) reported a live vulture sighting more than a year ago. Only 18.2% of interviewees reported having seen a live vulture during the survey year, 6.4% during the survey month and 1.8% during the survey week.

### Causes of vulture populations decline

**Fig. 5:**
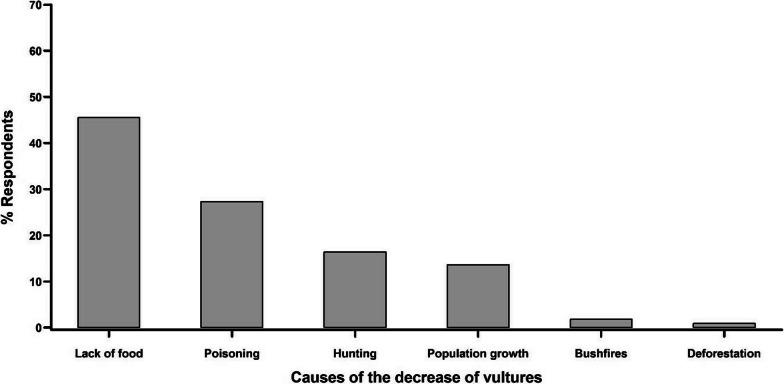
Reasons for the decline of vulture numbers according to the respondents

Numerous reasons for the decline in vultures were suggested by the interviewees. The most common reason (45.5%) was the reduction in food availability, followed by chemical pesticides (e.g. furadan) and tobacco poisoning (27.3%). Hunting, the growth of the human population, the occurrence of uncontrolled bush fires and the loss of natural habitats were also reported as sources of the decrease in vulture populations by 16.4%, 13.6%, 1.8% and 0.9% of those interviewed, respectively (Fig. 5).

### Uses of vultures and/or parts of vultures

#### Use frequency

**Fig. 6:**
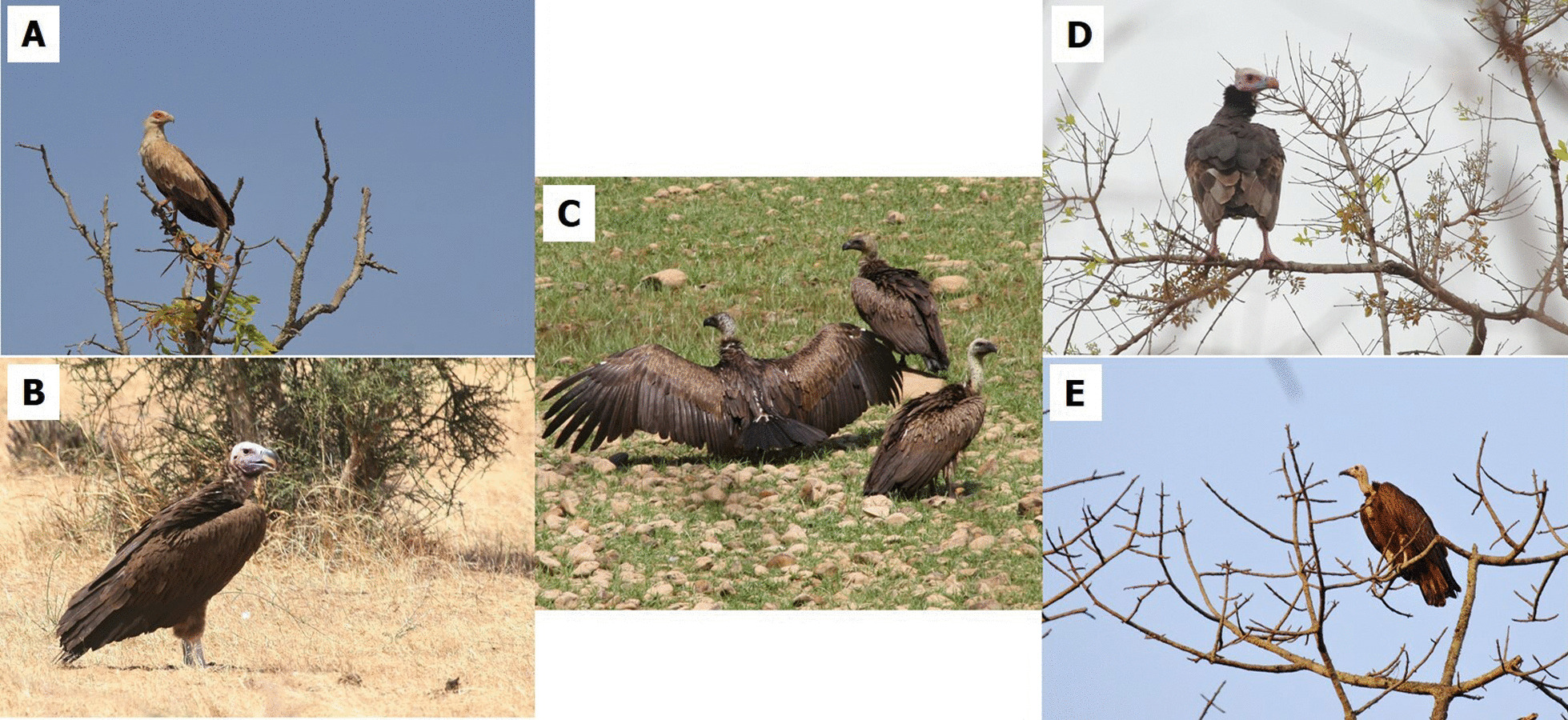
Picture of the five species of vulture used. **A** Palm-nut Vulture; **B** Lappet-faced Vulture; **C** White-backed Vulture; **D** White-headed Vulture; **E** Hooded Vulture

Sixteen and a half per cent of interviewees indicated that they had used vulture parts at least once, with no mention of the exact use made of them. It also emerged that the use of vultures was not related to the religion ($\chi^{{2}}_{{2}}$ = 0.761, *p* = 0.684), sex ($\chi^{{2}}_{{1}}$ = 0.004, *p* = 0.949) or age ($\chi^{{2}}_{{5}}$ = 1.892, *p* = 0.864) of the interviewees. Regarding how vultures and/or parts of vultures are obtained, a large proportion of respondents (66.4%) said they had no idea. Markets in nearby large villages or cities were identified by 19.1% of respondents and relatives and friends by 14.5%. The species used for these practises are White-headed Vulture, White-backed Vulture, Lappet-faced Vulture, Hooded Vulture and Palm-nut Vulture (Fig. 6).

### Use categories, consensus of use and use values by use categories and species

**Fig. 7:**
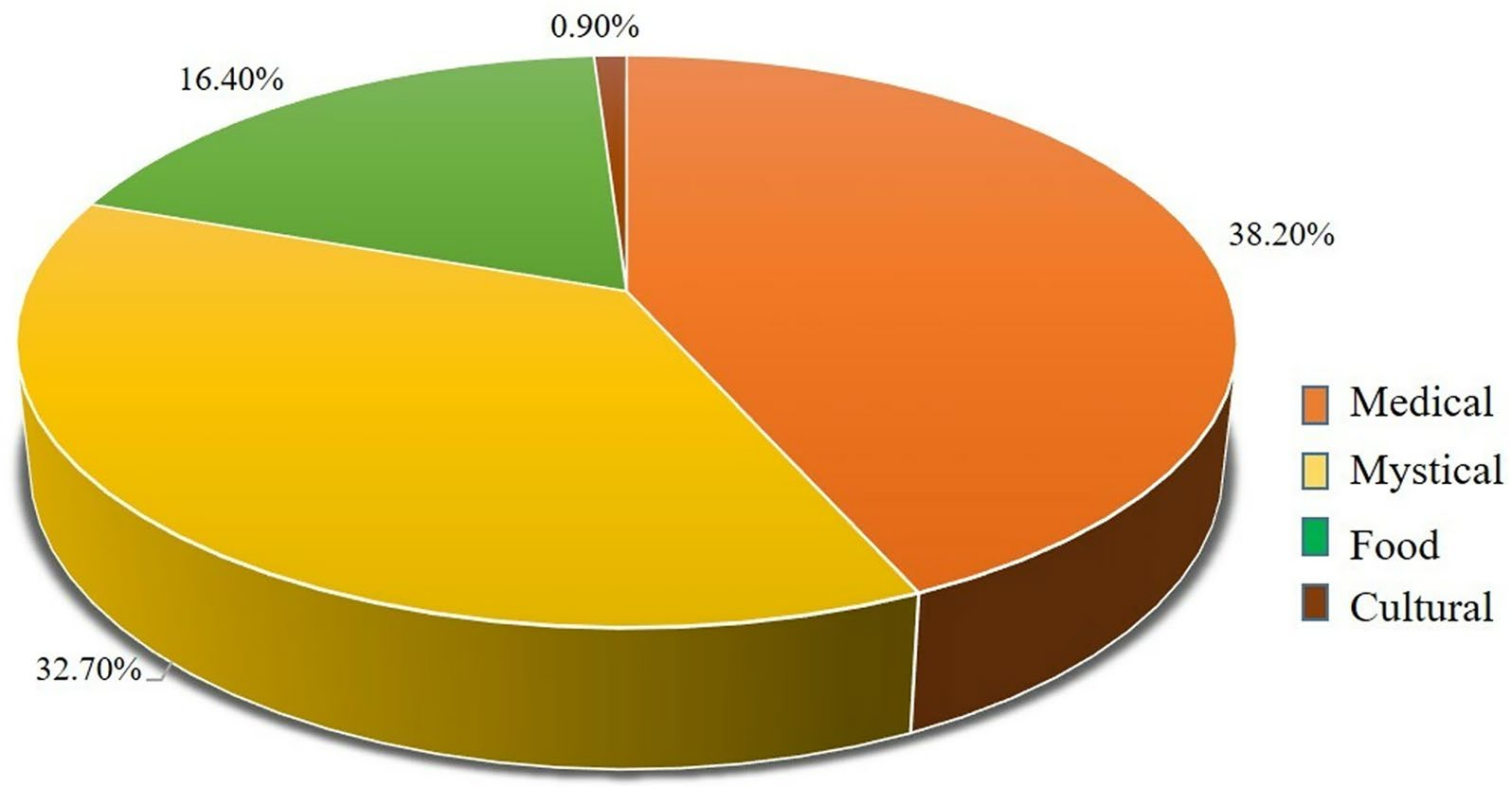
Use categories of vultures according to the uses mentioned by the respondents

The responses obtained on the use made of vultures or parts of vultures were classified into four categories (medicinal, mystic, food and cultural). Medicinal and mystical uses were cited by 38.2% and 32.7% of the interviewees, respectively (Fig. 7). The use of vultures as food was less frequently mentioned (16.4%) and only 2.72% mentioned the use of vultures for cultural purposes.

**Fig. 8:**
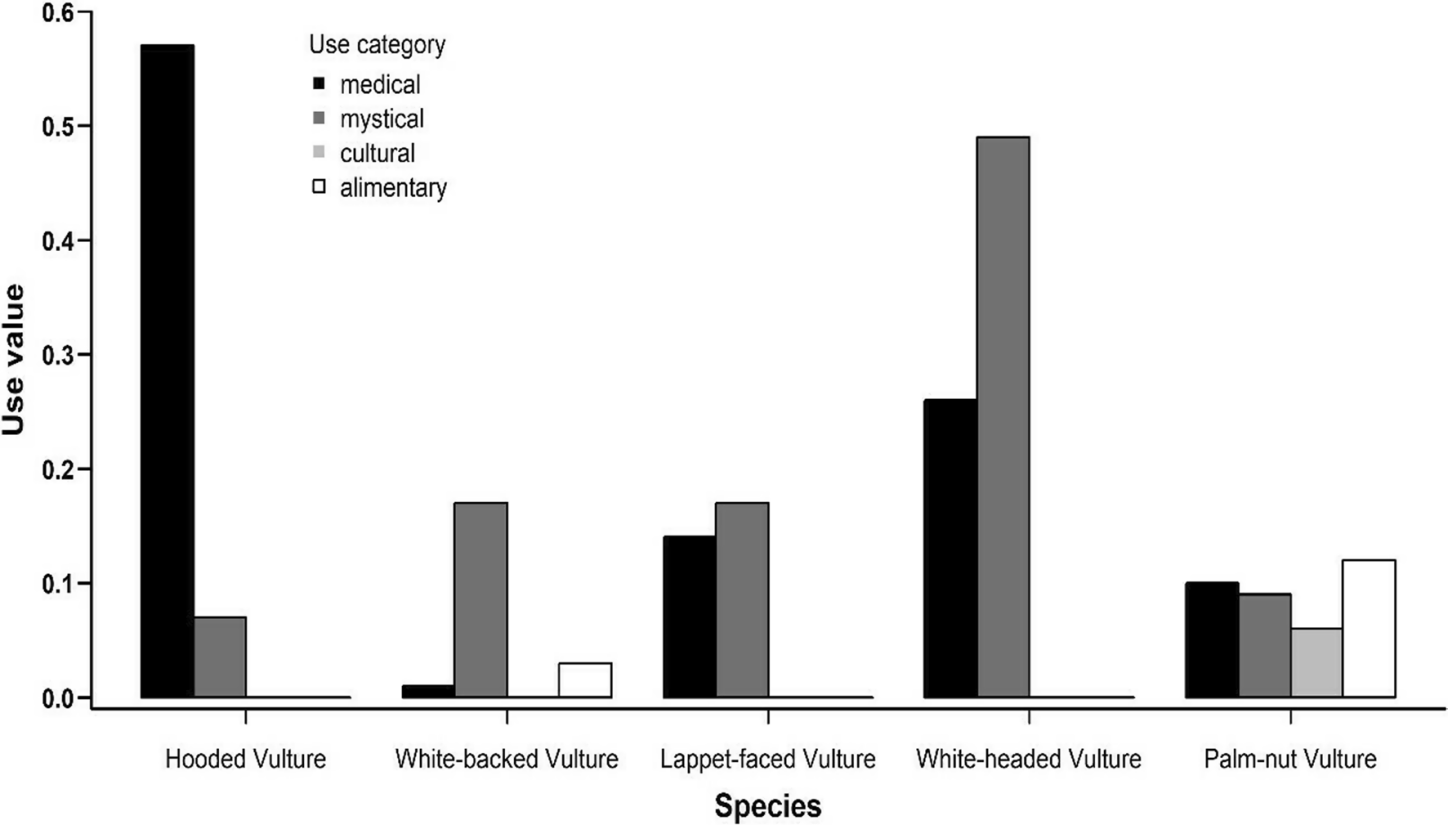
Species-specific use values for the different use categories

The use values indicated species-specific differences for the use categories (Fig. 8). Medical use was reported for all species, but the use values were especially high for the Hooded Vulture (0.57) and the White-headed Vulture (0.26). The latter species had also a high value (0.49) for mystical use, a category for which the other species reached only moderate values (0.07–0.17). The categories cultural and alimentary were only mentioned for the Palm-nut Vulture (Fig. 8).

**Fig. 9:**
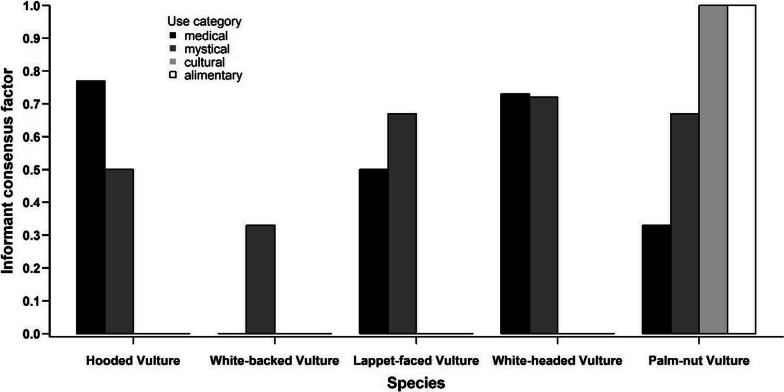
Species-specific consensus factors for the different use categories

The informant consensus factor reached the highest possible values in cultural and alimentary use for the Palm-nut Vulture (Fig. 9) again, because it was the only species mentioned in these two categories. There was a relatively high consensus across the respondents with respect to the use of parts of Hooded Vulture, Lapped-faced Vulture and White-headed Vulture with respect to medical and mystical uses, but with respect to medical use the informant consensus factor was lower for the Palm-nut Vulture (Fig. 9).

The different use categories vary in their importance across different vulture species (Table 3). UD (Table 1) was highest for medical use in Hooded Vulture and for mystical use for White-backed Vulture which reflects their importance in the respective categories. White-headed Vulture and Lappet-faced Vulture also reached relative high values in the latter category. An exception is the Palm-nut Vulture as UD reaches 0.32 in the category “alimentary” in which the only other mentioned species is the White-backed Vulture with a UD of 0.13 (Table 3).

**Table 3:** Use diversity value (UD) and use equitability value (UE) for the different use categories separated by species

Use category	Hooded vulture		White-headed vulture		White-backed vulture		Lappet-faced vulture		Palm-nut vulture	
	UD	UE	UD	UE	UD	UE	UD	UE	UD	UE
Medical	0.89	1.00	0.33	0.53	0.07	0.08	0.45	0.83	0.28	0.88
Mystical	0.11	0.13	0.63	1.00	0.80	0.90	0.55	1.00	0.24	0.75
Alimentary	–	–	–	–	0.13	0.17	–		0.32	1.00
Cultural	–	–	0.04	0.06	–	–	–	–	0.16	0.50

UE (Table 1) indicates the homogeneity of knowledge of the different use categories. A value of > 0.5 is reached in the category “medical” in Hooded Vulture, Lappet-faced Vulture, Palm-nut Vulture and just above this level in White-headed Vulture (Table 3). For these species, the respondents have an equal knowledge about their medical use (Table 1). In the category “mystical,” this value is reached in White-headed Vulture, White-backed Vulture, Lappet-faced Vulture and Palm-nut Vulture.

### Use of organs and excrements and their importance for medicinal and mystical use

Almost all organs of vultures are used for practises related to the different use categories.

**Fig. 10:**
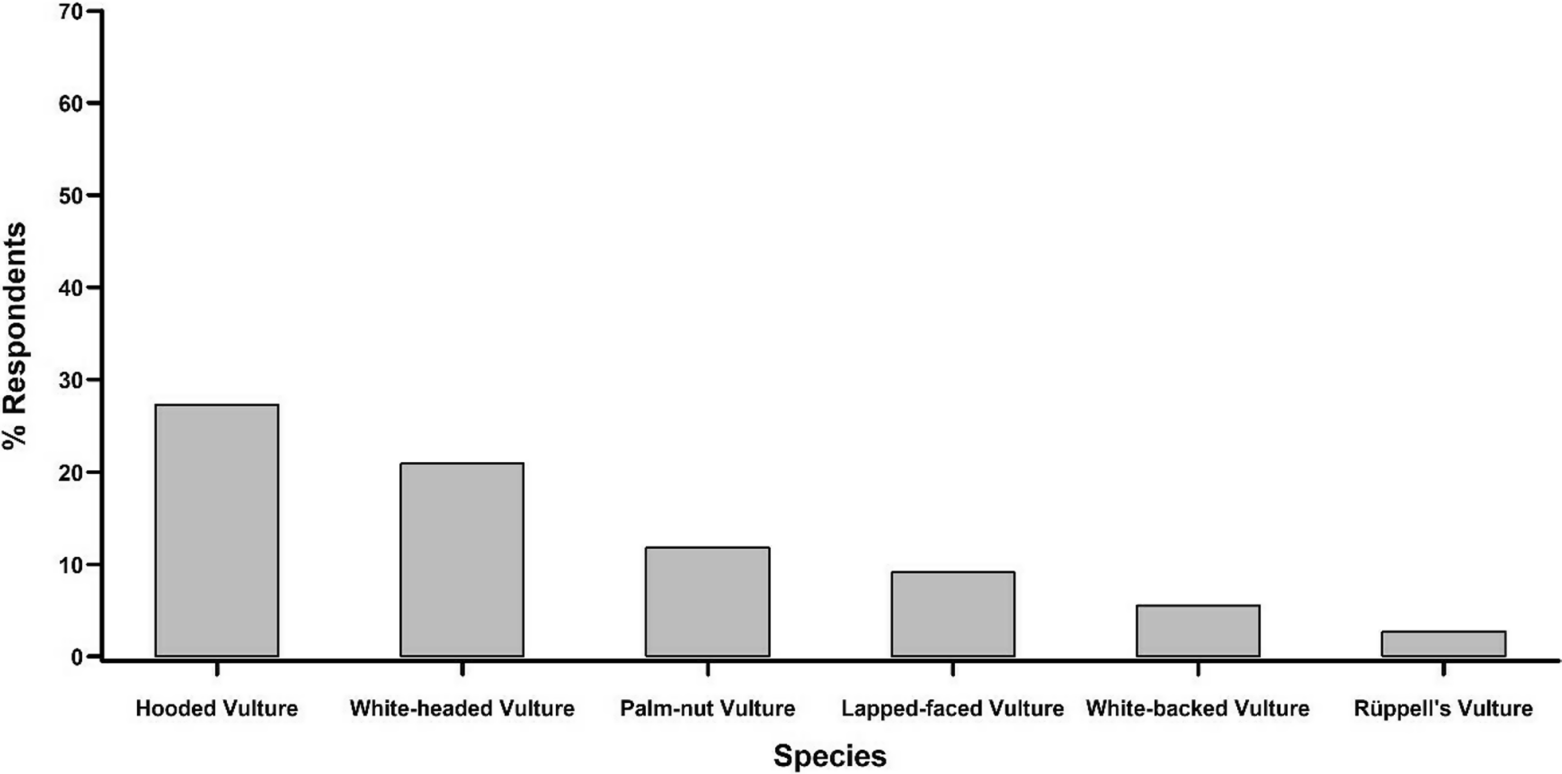
The species of vultures mostly used in traditional medicine according to the respondents

**Fig. 11:**
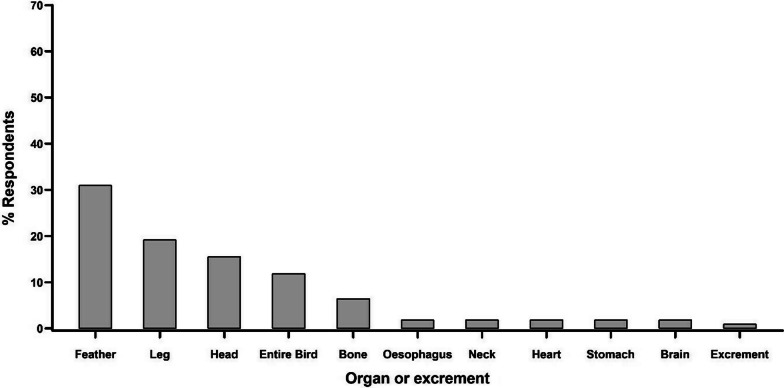
Use of different vultures organs according to respondents

In traditional medicine, the most sought-after vultures are the Hooded Vulture, followed by White-headed Vulture, Palm-nut Vulture, Lappet-faced Vulture, White-backed Vulture and Rüppell’s Vulture (Fig. 10). The parts of the vulture that are most frequently used (Fig. 11) are feathers (mentioned by 30.9% of the respondents), legs (19.1%), head (15.5%), the whole vulture (11.8%) and bones (6.4%). In addition, other parts that are sometimes mentioned were oesophagus (1.8%), neck (1.8%), heart (1.8%), stomach (1.8%), brain (1.8%) and excrements (0.9%). The citation frequencies and fidelity levels varied across organs and species (Table 4). However, both indices ranked high for head, feathers and foot/legs, although the absolute values differed across species. High values reached, e.g. feathers and head of Hooded Vulture and feathers of White-headed Vulture as well as of White-backed Vulture.

**Table 4:** Organs of different vulture species used in traditional medicine

Species	Organ	CF	FL	Use
Hooded vulture	Head	38.5	22.7	Makes women fertile
	Foot/leg	15.4	9.1	Remedy against poison
	Feather	84.6	50.0	Treats anaemia, tooth decay
	Heart	3.9	2.3	Remedy against pain
	Neck	3.9	2.3	Remedy against poison
	Oesophagus	3.9	2.3	Heals swollen feet
White-headed vulture	Foot/leg	6.1	3.7	Heals joint pain
	Feather	30.3	18.5	Treats madness
	Skin	3.0	1.9	Treats blurred vision
	Excrements	12.1	7.4	Treats earache
White-backed vulture	Heart	11.1	6.7	Treats heart problems
Lappet-faced vulture	Head	25.0	13.0	Treats heart problems
	Foot/leg	8.3	4.4	Treats heart problems
	Feather	33.3	17.4	Treats anaemia
	Bone	16.7	8.7	Treats anaemia
Palm-nut vulture	Head	14.3	8.0	Makes women fertile
	Foot/leg	21.4	12.0	Treats madness
	Feather	14.3	8.0	Treats food pain

Shown is the citation frequency (CF), the fidelity level (FL) and the use for each organ and the excrements separated by species

Organs of all vulture species are also used for several mystical practises, e. g. as protection against bad spells, increase success of a business, or for different aspects connected to hunting (Table 4). High citation frequencies (16.7–44.4) and fidelity levels (3.6–26.7) were found for feathers in all species except for Hooded Vulture (3.8/2.3) for which feathers ranked high in medical use (Table 4). Other organs that ranked high (CF > 10.0, Table 4) were head (White-backed Vulture), foot/leg (Palm-nut Vulture), the entire bird (Hooded Vulture, White-headed Vulture, Lappet-faced Vulture) and bone, eyes and blood in the White-backed Vulture. Organs that were less frequently mention were heart, stomach and brain (Table 5).

**Table 5:** Organs of different vulture species used for mystical practises

Species	Organ	CF	FL	Use
Hooded Vulture	Feather	3.8	2.3	Used to cast bad spells, makes a business grow, used by poachers to lure animals to water, used to gain supernatural strength
	Heart	3.8	2.3	Used by poachers to disappear in a dangerous situation
	Entire bird	11.5	6.8	Protection against evil spells, attracting customers to a business
White-headed Vulture	Head	27.3	8.2	Protection against bad spells, makes rain come
	Foot/leg	6.1	1.8	Protection against bad spells
	Feather	36.4	10.9	Used by hunters to fly away
	Stomach	6.1	1.8	Used to make poison
	Brain	3.0	0.9	Protection against bad spells
	Bone	6.1	1.8	Used to have a long life
	Entire bird	18.2	5.5	Attracting customers to a business, protection against bad spells
White-backed Vulture	Foot/leg	6.1	1.8	Protection against bad spells
	Feather	44.4	26.7	Settling disputes in one’s favour
	Bone	22.2	13.3	Attracting luck to oneself
	Eye	11.1	6.7	Used by hunters to locate animals
	Blood	11.1	6.7	Dedicating the clothing of traditional hunters
Lappet-faced Vulture	Head	8.3	4.4	Increases the number of guests in hotels and bars
	Foot/leg	8.3	4.4	Allows hunters to avoid missing their targets
	Feather	16.7	8.7	Makes a business grow
	Entire bird	14.3	1.8	Protection against bad spells
Palm-nut Vulture	Foot/leg	14.3	1.8	Promotes wish fulfilment
	Feather	28.6	3.6	Banishes evil spirits, protection against mystical attacks

Shown is the citation frequency (CF) and fidelity level (FL) for each organ separated by species

Only feathers were mentioned to be used for cultural ceremonies, i.e. for traditional dances and for hats of hunters. The citation frequencies were 6.1% for White-headed Vulture and 28.6% for Palm-nut Vulture with fidelity levels of 3.7% and 16.0%, respectively. Other species were not mentioned in connection with cultural practises.

According to the respondents, Palm-nut Vultures and White-backed Vultures are a source of food for the population surrounding CNP and all parts are used for consumption. The citation frequencies and fidelity levels were 57.1% and 32.0% for Palm-nut Vulture and 22.2% and 13.3% for White-backed Vulture, respectively.

### Attitudes towards vultures

**Fig. 12:**
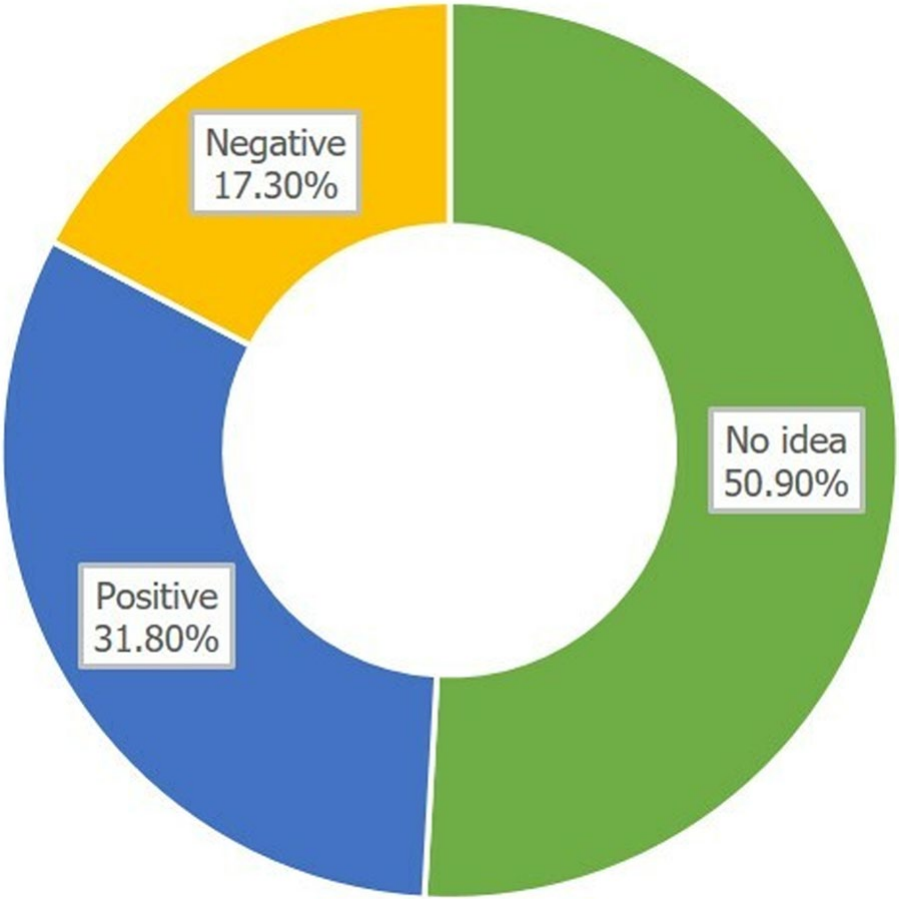
Attitude of the respondents towards vultures

A large number of respondents (50.9%, Fig. 12) were undecided about their perception of vultures, while 31.8% showed a positive perception towards them, indicating that the presence of a vulture in the village announces that one of the hunters will return successfully from hunting. On the other hand, 17.3% of the respondents indicated that they perceive vultures as a source of misfortune, because they are assumed to be the cause of the death of their livestock. The attitude towards vultures was neither linked to religion ($\chi^{{2}}_{{4}}$ = 1.403, *p* = 0.844), nor to the sex ($\chi^{{2}}_{{2}}$ = 1.089, *p* = 0.580) or the age ($\chi^{{2}}_{{{1}0}}$ = 18.079, *p* = 0.054) of the respondents.

## Discussion

### Knowledge of vultures by the interviewees

The majority of the interviewees claimed to be familiar with vultures. This suggests that vultures are, or at least were, part of their daily lives. However, their knowledge of the species is somewhat limited.

A high percentage of interviewees claimed to know Hooded Vultures which could reflect both, the former and relatively recent abundance of the species in villages in the north and east of the country [21, 45]. A similar high percentage of interviewees claimed to know the Palm Vulture, also abundant in the area, at least within the CNP, and with a very striking plumage. An unexpected result was the relatively high percentage of respondents who said they were familiar with the rare White-headed Vulture, Rüppell’s Vulture and Lappet-faced Vulture. This may be due to the fact that interviewees may tend to have an ideal image of vultures based on the photographs (only one per species) they were shown during the survey. The possibility of misidentification must be considered.

Local names of vulture species were well known, showing that vultures used to be part of the environment. These local names are still remembered by the local population, suggesting that there are still some vulture populations in the study area, although they are hardly observed outside CNP (own unpublished observations, this study). This also indicates that the population decline is relatively recent.

The local names attributed to vultures in the study area are very meaningful. Indeed, names like the “meat eater” and the “carcass eater” are attributed to Hooded Vulture, while “the white one” designates the palm vulture, whose body is essentially white. Furthermore, certain names reflect people’s familiarity with the ecology of some species, such as “the seed-eater” for the palm vulture, which feeds mainly on the fruits of various palm species [18]. The White-headed Vulture, which is able to hunt living prey on an almost regular basis [46], is known as the “hunter vulture”. Some local names with meanings difficult to understand have also been reported. Indeed, names such as “firebird”, “the profiteer” or “the bad one” are used to refer to some species by some interviewees. Such names might be a reference to the occurrence of seasonal bush fires [31], or to the fact that most species are scavengers.

The CNP was mentioned by almost half the people surveyed as the place where vultures are still found. The question is how these people know that the only refuge for vultures in their environment is the CNP. Indeed, this observation could indicate illegal activities in the park. Similarly, the foraging ecology of Palm-nut Vultures is reflected by sightings in villages during palm fruiting periods. It is also interesting to note that interviewees associate vulture sightings at the Bouna slaughterhouse with the dry season. These sightings must refer to Hooded Vultures, the only species regularly observed in villages in West Africa [18]. Mundy mentioned that part of the Hooded Vulture population in this African region may move from north to south, the rainy season being the main factor in this short migration [18]. However, Thiollay [19] does not mention any seasonality in the presence of Hooded Vultures in Côte d’Ivoire, but only a marked decline in White-backed Vulture populations during the rainy season.

### Perceptions about vulture populations sizes around the PNC

A decrease in numbers of vultures was reported by nearly all respondents (98.2%) and apparently at present vultures are a rare sight within and around villages. This is in line with results of monitoring programmes throughout West Africa [17, 47, 48] and correspondents with the results of interviews of local people in Burkina Faso [16], Nigeria [8, 49] and Ghana [25]. The pan-African trend [1] is recognised by local people throughout West Africa.

The putative reasons for the decline of vultures are diverse, but have in common that they are all linked to human activities. Direct persecution, i.e. poisoning and hunting combined, was mentioned by 43.7% of the respondents. This adds again to studies that suggested that persecution is the main threat for vultures in West Africa [10, 12]. More than a quarter of all respondents mentioned furadan (carbofuran) and tobacco poisoning, which seem to be common practise in other parts of West Africa too [13, 16, 25] along with other chemicals such as Gamaline 20 [50]. The results of this study suggest that a relatively high percentage of the inhabitants around CNP have a detailed knowledge about the methods of killing vultures, indicating that it is a widespread habit that is more or less openly practised. Interesting is also the high percentage (45.5%) of respondents that mention reduced food resources as a reason for decline, although the term “food resources” was not specified. We are not aware of any reduction of domestic animals (cows, goats, sheep) in and around villages. The latter may even increase with respect to CNP as there are reports of cattle within the park limits, especially in the northern areas [34, 35]. However, the connection between decreasing numbers of vultures and decreasing food resources could be based on the observation of decreasing populations of wild animals. At least large ungulates as well as other large mammals are almost non-existent outside CNP. Within the limits of CNP, poaching is widespread [34, 35]. The opinion of half of the respondents is likely to be indicative for an awareness of decreasing wildlife and suggests again that this decrease happened relatively fast and recently within the lifespan of the respondents or the former generation that could have told the respondents about the abundance of animals in former times. The reduction in carcass availability could be a problem for vultures in some parts of their range and it would be desirable to have more data on this problem [14].

### Use of vultures by the local populations

The majority of respondents indicated that vultures are used for different reasons such as medical, mystical or cultural practises (55.5%). That 16.4% claimed to have used vultures for themselves does not sound much, but considering 300,000 inhabitants in the vicinity of CNP [34] this would mean that almost 50,000 persons across religious borders, age and sex classes around CNP had used parts of vultures for various reasons. Although there is the flaw in this estimate that many of the inhabitants are much younger that the youngest respondent, it indicates a potentially high demand for vultures around one of their last strongholds in West Africa.

The knowledge about the use of vulture parts contrasts with the high proportion (66.4%) of people who claimed that they have no knowledge where those parts could be made available. Respondents talked openly about the use of vultures, stalls with parts of animals including vultures are found on many if not most markets and are not hidden, in one village a traditional healer showed a small house to the first author of this study and called it his magazine. Although the author was not allowed to have a look inside other inhabitants of the village must know its content and traditional healers are found in many villages. Therefore, we assume that the high percentage of people claiming not to know how parts of vultures could be made available is likely linked to their knowledge that this trade is illegal or that vultures are taken in CNP.

With respect to conservation, the people’s attitude towards vultures is important. However, although almost one-third of the respondents showed a positive attitude towards vultures, this was linked to hunting. As CNP is the only area with considerable populations of large mammal species in the region, hunting probably often means poaching in CNP. Vultures are not valued by their beneficial ecosystem services [51–53], but because they are harbingers of successful hunting/poaching. In contrast, a distinct proportion of respondents (17.3%) have a negative attitude towards vultures because they believe that vultures cause the death of their life stock. This belief is most likely rooted in the regular observation of vultures near/on dead animals and the signs of an important ecosystem service, the fast removal of carcasses that could spread diseases [51, 53], are erroneously interpreted for the disadvantage of vultures. This contrasts with findings in KwaZulu-Natal, South Africa, where vultures are valued for the fast removal of cattle carcasses [54].

There are differences in the potential demand for certain species, use categories and organs of vultures. Vultures are most sought for medical and mystical uses. Other studies suggested that the combined categories, that are also known under the terms “traditional medicine” and “belief-based use” [11, 13, 16, 55], are the main reason for the continent-wide decline of vultures and an important threat for local vulture populations [8, 12, 13, 15, 56]. We do not know to which extent the results of our study are representative for the entire country, but a respective study is under way [57]. In Abidjan, Côte d’Ivoire’s economic capital c. 360 km south of CNP, vultures are sought for and a stall owner informed two of us (AAA, VS), that even entire vultures are quickly sold when available. As vultures do not occur in southern Côte d’Ivoire regularly at least for some decades [3], vultures on markets in Abidjan must originate either from the north of the country, e. g. from CNP, or obtained through trans-border trade [13, 16, 57]. However, like in other West African countries, there is a demand for vultures in Côte d’Ivoire that drives killing and trading critically endangered species [57].

There is some consensus about the use of species and organs among the respondents indicating a common perception across religious and cultural borders. The most sought vulture species for traditional medicine is Hooded Vulture [55]. This may be linked to the fact that this species is mostly associated with settlements and therefore, the most frequently encountered and available species. Additionally, there may be some uncertainty in the identification of the rarer species (see above) which renders it difficult to interpret answers about the use of certain species. Surprising, and not in line with other studies, is that the most sought parts of vultures are feathers and legs and that the head is mentioned only at the third place. The latter or the brain is mentioned as the most sought body part in Ghana [58], Nigeria [26, 49] and for KwaZulu-Natal, South Africa [59]. New is here, that some organs are especially mentioned such as oesophagus, heart or stomach, although no special remedy was linked to these organs.

Despite the great cultural and religious diversity in West Africa [60], there are parallels of our results from a restricted geographical area to other studies, even in other parts of Africa. This includes attracting good luck [11, 13, 55, 59], protection against evils spells [11, 13, 55, 58], success in economic activities/gaining money [8, 25, 59], acquiring supernatural strength [11]. Interestingly, and in contrast to other parts of Africa [7, 11, 59], no clairvoyant powers were mentioned.

That vultures are consumed for food (16.4% of respondents) seems to be odd, regarding their usual habits and, in some areas, their negative perception [8, 25]. Nevertheless, Thiollay [19] mentioned that hunting Hooded Vultures for food is the reason for the general decline in Côte d’Ivoire and Ogada and Buij [6] mentioned reports of the slaughter of Hooded Vultures for food in Burkina Faso. When J.-M. Thiollay was specifically asked about his statement [19] by one of us (VS), he mentioned restaurants in Bouaké, the second largest city in Côte d’Ivoire, that had vultures on their menu in the 1970s (J.-M. Thiollay, pers. comm.). That not all vultures consumed as food were deliberately eaten as such was mentioned to VS in the late 1990s. A local from a village at the fringe of CNP mentioned the custom of chopping heads and feet off dead vultures selling the remaining corpse as turkeys. This is in line with Ogada and Buij [6] (mixing of vulture meet with chicken meet in Burkina Faso) and Gbogbo [17] (selling vulture meet to unsuspecting customers in Ghana). Some authors even went so far to mention the possibility that the use as food could be the main cause for the disappearance of Hooded Vultures from many towns and villages [14, 50]. With respect to the high prices paid for vultures and parts of vultures nowadays [8, 16, 17, 25] and that even other raptor species, such as Black Kites *Milvus sp*., get head-feathers plucked and sold as vultures [61], this practise is likely to be abandoned for economic reasons now [6].

## Conclusions

This study aimed to analyse the human pressures on vultures originating from cultural attitudes and uses of all or part of these organisms, in the vicinity of one of their last major refuges in Côte d’Ivoire; the Comoé National Park (CNP). It revealed that a number of uses are made of vultures and parts of them by local populations surrounding the CNP. Many of these uses are related to cultural beliefs and practises, highlighting the importance of taking into account the interaction between cultural practises, as well as traditional medicine and the physical environment, when developing and implementing sustainable conservation strategies for these raptors. For a better understanding of the threats vultures face in and around the CNP, a socioeconomic assessment of the trade of poached vultures as well as a national, sub-regional and regional overview of these illegal practises would be useful.

### Supplementary Information

**Additional file 1**. The file presents the proportions of respondents' professions and religions by gender and age.

## Data Availability

The datasets used and/or analysed during the current study are available from the corresponding author on reasonable request.

## Abbreviations

CNP: Comoé National Park
CMS: Convention of migratory species
CITES: Convention on International Trade in Endangered Species
NABU: International Foundation for Nature
OIPR: Office Ivorian of Parks and Reserves
UV: Use value
FIC: Informant consensus factor
UD: Use diversity index
UE: Use equitability value
CF: Citation frequency
FL: Fidelity level

## References

[1] Ogada D, Shaw P, Beyers RL, Buij R, Murn C, Thiollay J-M, Beale CM, Holdo RM, Pomeroy D, Baker N, Krüger SC, Botha A, Virani MZ, Monadjem A, Sinclair ARE (2016). Another continental vulture crisis: Africa’s vultures collapsing toward extinction. Conserv Lett.

[2] Botha AJ, Andevski J, Bowden CGR, Gudka M, Safford RJ, Tavares J, Williams NP (2017). Multi-species action plan to conserve African-Eurasian vultures. CMS Raptors MOU Tech Publ.

[3] Thiollay J-M (1975). Les rapaces d’une zone de contact savane-forêt en Côte-d’Ivoire: densite, dynamique et structure du peuplement. Alauda.

[4] Cook AW, Mundy PJ (1980). Rüppell’s griffon vulture at Kotorkoshi, Nigeria. Malimbus.

[5] Thiollay J-M (2006). The decline of raptors in West Africa: long-term assessment and the role of protected areas. Ibis.

[6] Ogada DL, Buij R (2011). Large declines of the Hooded Vulture *Necrosyrtes monachus* across its African range. Ostrich.

[7] Wacher T, Newby J, Houdou I, Harouna A, Rabeil T (2013). Vulture observations in the Sahelian zones of Chad and Niger. Bull Afr Bird Club.

[8] Williams MM, Ottosson U, Tende T, Deikumah J (2021). Traditional belief systems and trade in vulture parts are leading to the eradication of vultures in Nigeria: an ethno-ornithological study of north-central Nigeria. Ostrich.

[9] Henriques M, Buij R, Monteiro H, Sá J, Wambar F, Tavares JP, Botha A, Citegetse G, Lecoq M, Catry P, Ogada D (2020). Deliberate poisoning of Africa’s vultures. Science.

[10] Nikolaus G (2001). Bird exploitation for traditional medicine in Nigeria. Malimbus.

[11] Saidu Y, Buij R (2013). Traditional medicine trade in vulture parts in northern Nigeria. Vulture News.

[12] Buij R, Nikolaus G, Whytock R, Ingram DJ, Ogada D (2016). Trade of threatened vultures and other raptors for fetish and bushmeat in West and Central Africa. Oryx.

[13] Adjakpa JB, Tchabi A, Ogouvide FT (2002). Oiseaux utilisés en pharmacopée traditionnelle au Bénin. Malimbus.

[14] Rondeau G, Thiollay J-M (2004). West African vulture decline. Vulture News.

[15] Manja WM, Tende T, Ottosson U, Deikumah JP (2021). Abundance, distribution, and threats affecting Hooded Vultures in north-central Nigeria. J Res For Wildl Environ.

[16] Daboné C, Ouéda A, Thompson LJ, Adjakpa JB, Weesie PDM (2022). Trade in vulture parts in West Africa: Burkina Faso may be one of the main sources of vulture carcasses. Bird Conserv Int.

[17] Gbogbo F, Roberts JST, Awotwe-Pratt V (2016). Some important observations on the populations of Hooded Vultures *Necrosyrtes monachus* in urban Ghana. Int J Zool.

[18] Mundy P, Butchart D, Ledger J, Piper S (1992). The vultures of Africa.

[19] Thiollay J-M (1985). The birds of Ivory Coast: status and distribution. Malimbus.

[20] Bouet G, Millet-Horsin H (1916). Liste des oiseaux recueillis ou observés à la Côte d'Ivoire en 1906–1907 et en 1913–1914. Revue Francaise d’Ornithologie.

[21] Thiollay J-M (1975). Les rapaces des parcs nationaux de Côte d'Ivoire. Analyse du peuplement. L’Oiseau R.F.O..

[22] Salewski V (2021). Vulture numbers and densities in a large protected savannah in West Africa. Acta Oecol..

[23] Salewski V (2017). Comoé National Park - a refuge for critically endangered vulture species in West Africa. Vulture News.

[24] Onoja JD, Tende T, Omotoriogun TC, Ottosson U, Manu SA, Mwansat GS (2014). Raptors in Yankari Game reserve and surrounding unprotected area, Nigeria. Malimbus.

[25] Deikumah JP (2020). Vulture declines, threats and conservation: the attitude of the indigenous Ghanaian. Bird Conserv Int.

[26] Awoyemi SM (2014). Vulture declines in West Africa: investigating the scale and (socioeconomic) drivers of the trade in vulture parts for traditional medicine. MPhil-thesis.

[27] Weladji R, Moe S, Vedeld P (2003). Stakeholder attitudes wildlife policy and Bénoué wildlife conservation area, North Cameroon. Environ Conserv.

[28] Adams WM, Aveling R, Brockington D, Dickson B, Elliott J, Hutton J, Roe D, Vira B, Wolmer W (2004). Biodiversity conservation and the eradication of poverty. Science.

[29] Baldus R, Kibonde B, Siege L (2003). Seeking conservation partnerships in the Selous game reserve, Tanzania. Parks.

[30] Epanda MA, Fotsing AJM, Bacha T, Frynta D, Lens L, Tchouamo IR, Jef D (2019). Linking local people’s perception of wildlife and conservation to livelihood and poaching alleviation: a case study of the Dja biosphere reserve, Cameroon. Acta Oecol.

[31] Poilecot P (1991). Un écosystème de savane soudanienne: le Parc National de la Comoé (Côte d’Ivoire).

[32] Porembski S (1991). Beiträge zur pflanzenwelt des comoé-nationalparks (Elfenbeinküste). Natur Mus.

[33] Salewski V (2000). The birds of Comoé National Park, Ivory Coast. Malimbus.

[34] OIPR (Office Ivoirien des Parcs et Reserves). Plan d’amenagement et de gestion du Parc National de la Comoé. Site du Patrimoine mondial et d’une Réserve de biosphère. Abidjan; 2015.

[35] Henschel P, Azani D, Burton C, Malanda G, Saidu Y, Sam M, Hunter L (2010). Lion status updates from five range countries in West and Central Africa. CATnews.

[36] British Sociological Association. Statement of ethical practice for the British sociological association. 2017. Available at: www.britsoc.co.uk/ethics

[37] Philips O, Gentry AH, Reynel C, Wilkin P, Galvez-Durand C (1994). Quantitative ethnobotanyand Amazonian conservation. Conserv Biol.

[38] Sop TK, Oldeland J, Bognounou F, Schmiedel U, Thiombiano A (2012). Ethnobotanical knowledge and valuation of woody plants species: a comparative analysis of three ethnic groups from the sub-Sahel of Burkina Faso. Environ Dev Sustain.

[39] Trotter RT, Logan MH, Etkin NL (1986). Informant consensus: a new approach for identifying potentially effective medicinal plants. Plants in indigenous medicine and diet.

[40] Atta ACJ, Soulemane O, Kadjo B, Kouadio YR (2021). Some uses of the African buffalo *Syncerus caffer* (Sparrman, 1779) by the populations living around the Comoé National Park (North-East Ivory Coast). J Anim Plant Sci.

[41] Byg A, Balslev H (2001). Diversity and use of palms in Zahamena, eastern Madagascar. Biodivers Conserv.

[42] Awo H, Chaffra S, Yabi F, Lougbegnon T, Djondo M, Tente B (2020). Ethno-zoological study and forms of use of Trichechus senegalensis in Southern Benin. Moroc J Agron Vet Sci.

[43] Mouzoun S. Écology et cannaissances ethnozoologiques du porc-épic a crête (Hystrix cristata Linnaeus, 1758) dans les réserves de biosphère de la Pendjari et du W au Bénin. Ph-D Thesis, Université d’Abomey-Calavi, Bénin; 2018.

[44] Ugulu I (2012). Fidelity level and knowledge of medicinal plants used to make therapeutic Turkish baths. Stud Ethno-Med.

[45] Demey R, Fishpool LDC (1991). Additions and annotations to the avifauna of Côte d’Ivoire. Malimbus.

[46] Murn C (2014). Observations of predatory behavior by white-headed vultures. J Raptor Res.

[47] Thiollay J-M (2006). Large bird declines with increasing human pressure in savannah woodlands (Burkina Faso). Biodivers Conserv.

[48] Nosazeogie E, Tende T, Monadjem A (2018). Hooded Vultures (*Necrosyrtes monachus*) nearly extirpated from Edo State, Nigeria: a report on the avian scavenger community. Ostrich.

[49] Owolabi BA, Odewumi SO, Agbelusi EA (2020). Perceptions on population decline and ethno-cultural knowledge of Hooded Vulture (*Necrosyrtes monachus*) in Southwest States of Nigeria. Vulture News.

[50] Tende T, Ottosson U (2008). The current status of vultures in Yankari game reserve, Nigeria. Vulture News.

[51] Ogada DL, Torchin ME, Kinnaird MF, Ezenwa AVO (2012). Effects of vulture declines on facultative scavengers and potential implications for mammalian disease transmission. Conserv Biol.

[52] Gangoso L, Agudo R, Anadón JD, de la Riva M, Suleyman AS, Porter R, Donázar JA (2012). Reinventing mutualism between humans and wild fauna: insights from vultures as ecosystem services providers. Conserv Lett.

[53] Moléon M, Sánchez-Zapata JA, Margalida A, Carrete M, Owen-Smith N, Donázar JA (2014). Humans and scavengers: the evolution of interactions and ecosystem services. Bioscience.

[54] Manqele NS, Selier SAJ, Taylor J, Downs CT (2023). Vulture perceptions in a socio-ecological system: a case study of three protected areas in KwaZulu-Natal, South Africa. J Ornithol.

[55] Sodeinde SO, Soewu DA (1999). Pilot study of the traditional medicine trade in Nigeria. Traffic Bulletin.

[56] Beilis N, Esterhuizen J (2005). The potential impact on Cape Griffon Gyps coprotheres populations due to the trade in traditional medicine in Maseru. Lesotho Vulture News.

[57] Asso AA, Koné NA, Salewski V. Socio-economic value of poached vultures and the content of potential cross-border trade affecting the diversity and abundance of these raptors in Côte d'Ivoire. Article in preparation; 2024.

[58] Boakye MK, Wiafe ED, Ziekah MY (2019). Ethnomedicinal use of vultures by traditional medicinal practitioners in Ghana. Ostrich.

[59] Manqele NS, Selier SAJ, Downs CT (2023). The ethnomedicinal use of vultures by traditional health practitioners in KwaZulu-Natal, South Africa. J Ornithol.

[60] Gervais-Lambony P, Nyassogbo GK. Les Etats-nations face à l’intégration régionale en Afrique de l'Ouest. Le cas du Niger. KARTHALA editions; 2007.

[61] Nikolaus G, Schuchmann K-L (2011). The fetish culture in West Africa: An ancient tradition as a threat to endangered birdlife?. Tropical vertebrates in a changing world Bonner Zoologische Monographien.

