# Supplementary material for: Cultural attitudes and human pressure towards vultures around the Comoé National Park, Côte d’Ivoire (West Africa)
Source: J Ethnobiol Ethnomed. 2024 Feb 29;20:30. doi: 10.1186/s13002-024-00657-0 (PMC10903046; doi:10.1186/s13002-024-00657-0)
Supplement: Supplementary file 1 — Additional file 1. The file presents the proportions of respondents' professions and religions by gender and age. [file 13002_2024_657_MOESM1_ESM.docx]

| **Gender** | **Age (years)** | **Profession** | | | | **Religion** | | | **Total by gender** |
| --- | --- | --- | --- | --- | --- | --- | --- | --- | --- |
|  |  | Farmer | Housewife | Shepherd | Without occupation | Animist | Muslim | Christian |  |
| Male | >60 | 25 (28.4%) | 0 | 0 | 0 | 13 (14.8%) | 9 (10.2%) | 3 (3.4%) | 25 (28.4%) |
|  | 51-60 | 14 (15.9%) | 0 | 0 | 0 | 4 (4.5%) | 9 (10.2%) | 1 (1.1%) | 14 (15.9%) |
|  | 41-50 | 24 (27.3%) | 0 | 0 | 0 | 14 (15.9%) | 8 (9.1%) | 2 (2.3%) | 24 (27.3%) |
|  | 31-40 | 15 (17.0%) | 0 | 0 | 0 | 10 (11.4%) | 4 (4.5%) | 1 (1.1%) | 15 (17.0%) |
|  | 21-30 | 7 (8.0%) | 0 | 1 (1.1%) | 1 (1.1%) | 8 (9.1%) | 1 (1.1%) | 0 | 9 (10.2%) |
|  | <21 | 1 (1.1%) | 0 | 0 | 0 | 1 (1.1%) | 0 | 0 | 1 (1.1%) |
| Female | >60 | 0 | 7 (31.8%) | 0 | 0 | 4 (18.2%) | 1 (4.5%) | 2 (9.1%) | 7 (31.8%) |
|  | 51-60 | 0 | 4 (18.2%) | 0 | 0 | 1 (4.5%) | 3 (13.6%) | 0 | 4 (18.2%) |
|  | 41-50 | 0 | 7 (31.8%) | 0 | 0 | 2 (9.1%) | 3 (13.6%) | 2 (9.1%) | 7 (31.8%) |
|  | 31-40 | 0 | 2 (9.1%) | 0 | 0 | 0 | 0 | 2 (9.1%) | 2 (9.1%) |
|  | 21-30 | 0 | 2 (9.1%) | 0 | 0 | 0 | 1 (4.5%) | 1 (4.5%) | 2 (9.1%) |
|  | <21 | 0 | 0 | 0 | 0 | 0 | 0 | 0 | 0 |

**Table Supplementary:** Respondents' occupation and religion by gender and age.
